# A Comparison between Temperature-Controlled Laminar Airflow Device and a Room Air-Cleaner in Reducing Exposure to Particles While Asleep

**DOI:** 10.1371/journal.pone.0166882

**Published:** 2016-11-29

**Authors:** Michal P. Spilak, Torben Sigsgaard, Hisamitsu Takai, Guoqiang Zhang

**Affiliations:** 1 Danish Building Research Institute, Aalborg University, Copenhagen, Denmark; 2 Aarhus University, Department of Public Health—Institute of Environmental and Occupational Medicine, Aarhus C, Denmark; 3 Aarhus University; Department of Engineering—Engineering Centre Bygholm, Horsens, Denmark; 4 Aarhus University, Department of Engineering—Fluid Dynamics and Building Ventilation, Aarhus C, Denmark; Universite de Bretagne Occidentale, FRANCE

## Abstract

People spend approximately one third of their life sleeping. Exposure to pollutants in the sleep environment often leads to a variety of adverse health effects, such as development and exacerbation of asthma. Avoiding exposure to these pollutants by providing a sufficient air quality in the sleep environment might be a feasible method to alleviate these health symptoms.

We performed full-scale laboratory measurements using a thermal manikin positioned on an experimental bed. Three ventilation settings were tested: with no filtration system operated, use of portable air cleaner and use of a temperature-controlled laminar airflow (TLA) device. The first part of the experiment investigated the air-flow characteristics in the breathing zone. In the second part, particle removal efficiency was estimated. Measured in the breathing zone, the room air cleaner demonstrated high turbulence intensity, high velocity and turbulence diffusivity level, with a particle reduction rate of 52% compared to baseline after 30 minutes. The TLA device delivered a laminar airflow to the breathing zone with a reduction rate of 99.5%. During a periodical duvet lifting mimicking a subject’s movement in bed, the particle concentration was significantly lower with the TLA device compared to the room air cleaner. The TLA device provided a barrier which significantly reduced the introduction of airborne particles into the breathing zone. Further studies should be conducted for the understanding of the transport of resuspended particles between the duvet and the laying body.

## Introduction

The sleep environment is an important yet understudied environment defined as space encompassing a mattress, bedding and a volume of space above these items. There are several defining parameters that make this environment unique from other indoor environments. This includes a prolonged exposure period people spend sleeping making up nearly one third of their lives [1, 2], a potential for higher exposure due to the close proximity of the pollutant source to the breathing zone (BZ) and diversity of pollutants including both, gaseous-phase pollutants and particulate matter (PM). Airborne PM present in the sleep environment is derived from outdoor air and from a variety of indoor sources including human-related activities, such as cooking and cleaning, and various biological sources, e.g. mold, fungal spores, pet and house dust-mite (HDM) allergens. Exposure to PM commonly found in the sleep environment has been consistently associated with various health outcomes, mainly development and further exacerbation of asthma [3, 4].

In 2000, the Committee on the Assessment of Asthma and Indoor Air of the Institute of Medicine (IOM) reviewed and summarized the scientific evidence for relationships between indoor air exposures and the exacerbation and development of asthma [5]. A recent review, incorporating evidence reported since 2000, further increased the strength of evidence linking many indoor factors to the exacerbation of asthma [6]. They concluded that multiple indoor exposures merit increased attention to prevent exacerbation of asthma. For exacerbation of asthma, there are sufficient evidence showing a causal relationship for HDM, cat and cockroach allergen exposure in individuals sensitized to these allergens [6, 7].

Further evidence for the causality of exposure to allergens exacerbating asthma comes from studies that has shown that if major allergen reductions (i.e., 95 percent reduction of mite allergen) can be achieved this can reduce both symptoms and bronchial reactivity. For example, moving patients to a hospital room or sanatorium has been consistently effective [8, 9, 10]. These units generally have very low levels of mites (i.e., less than 20 mites/g of dust) and mite allergen (less than 0.4 μg of Der p 1/g). Thus, avoidance of exposure to allergens should be an important component of the management of asthma and allergic diseases. The transportation mechanism of PM in the sleep environment is simple. First, the dust accumulated on the surface of mattresses, duvets pillows and bedding sheets is resuspended due to human-induced movements on the bed [11]. During the resuspension process the particles become airborne via applying one or more external forces, e.g. air burst from beneath the mattress surface, thermal plume around the human body, draft caused by the ventilation system and air flows generated by the respiratory process [12, 13, 14]. The risk of exposure to these particles is their further transport into the BZ where they can be inhaled by the sleeping person. In general, the amount of PM in the BZ that may be inhaled is influenced by several factors: body position of the sleeping person [14], strength of the thermal plume around human body [15, 16, 17] and the size of the particle [11].

Fan-forced HVAC systems commonly used in residential buildings can deliver relatively large amounts of air. However, the central distribution mixes the clean, filtered air with the ambient room air. Thanks to the mixing effect, the PM concentration in the supply air when approaching the BZ is almost equal to the concentration in the ambient air. Portable air cleaners (also known as air purifiers) are often considered as an effective solution because of high particle-removal efficiency ranging from 60 to 80% and non-installation requirements [12, 18]. Yet, the filtered air distributed by the portable air cleaner behaves similarly to the air delivered by fan-forced HVAC systems. In addition, the air momentum creates strong drafts which might lead to another negative effect–resuspension of particles deposited on bedroom surfaces (e.g. furniture and floor) and delivery of the resuspended particles into the BZ. The resuspension of the mattress dust and subsequent transport has been investigated by [11] and [12]. Both studies found the intake factor, i.e. theoretical amount of airborne particles inhaled during the sleep period, to be significantly higher compared to other indoor environments. This might explain why many studies focusing on the impact of air cleaning found little or no effect on participants’ perennial asthma [19, 20, 21].

Recently, a new environmental control method–temperature-controlled laminar air flow (TLA)–has been introduced [22]. This method is based on local downward laminar air delivery of cleaned, air-conditioned air of low speed from overhead supply nozzle. The cooler denser air falls and acts counter to the upward direction of the free convection flow (FCF) generated by the human head of a sleeping person. Because the laminar air flow of the supply air minimizes the mixing with the outer air, and the air is delivered locally straight to the BZ, a significantly lower amount of air is needed to deliver clean air to the BZ compared to traditional HVAC system or portable air cleaners. Four studies have already tested the performance of TLA device. [22] investigated the effect of TLA treatment on reduction of particle and cat allergen exposure. The clinical effectiveness of TLA treatment in patients suffering from persistent allergic asthma has been investigated in three clinical studies [23, 24, 25]. [23] and [24] conducted placebo controlled studies showing a significant improvement in health-related quality of life and a significant reduction in airway inflammation with the TLA treatment as compared to placebo in patients with moderate to severe asthma allergic to at least one perennial indoor allergen. [25] conducted a pre-post observational study over 12 months in patients with severe to very severe asthma showing a significant reduction in asthma exacerbations and related health-care utilization during the 12 months with the TLA treatment as compared to the year before. The study by [24] also confirmed the significant impact of TLA treatment on the BZ air quality.

However, further description of air flow characteristics in the breathing zone under TLA treatment has not yet been investigated. Therefore the objectives of this study were to i) investigate the impact of different ventilation techniques (including TLA treatment) on air flow characteristics in the BZ ii) compare the particle-removal effectiveness of the TLA device and portable room air cleaner. Both objectives were performed using a thermal manikin in a full-scale experimental chamber.

## Methods and Material

### Experimental design

Full-scale experiments were conducted in an environmental chamber with a thermal, full-sized manikin. The investigation consisted of two separate experimental studies. In the following text the first study is referred to as an “Airflow Characteristics Study (ACS)” and the second study is referred to as “Particle Concentration Study (PCS)”. The aim of the ACS was to describe air flows in the BZ in order to explore the fundamental mechanisms of particle exposure and the PCS assessed the particle-removal efficiency of different ventilation strategies in the BZ. In the ACS three ventilation settings were tested i) without any ventilation operating (ventilation setting A) ii) with the use of a portable air cleaner (ventilation setting B) and iii) with the use of a TLA device (ventilation setting C).

**Fig 1:**
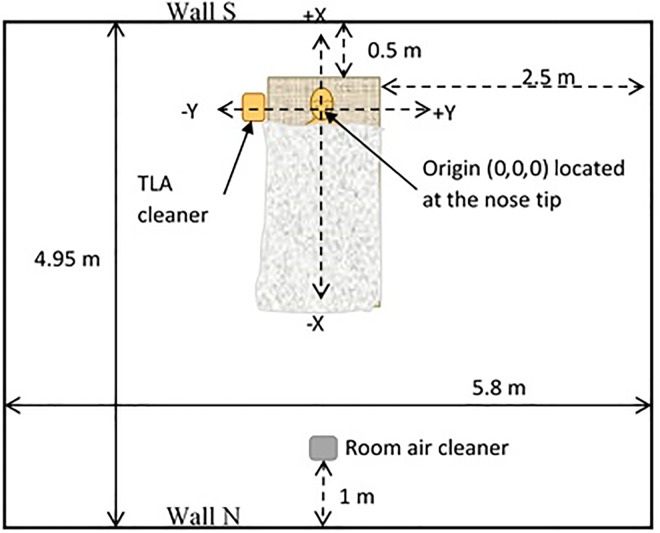
Experimental chamber setup.

For all tested ventilation settings (i.e. setting A, setting B and setting C) one velocity sensor was positioned 8 cm above the manikin’s nose tip (Fig 1). To further explore the TLA treatment an additional set of measurements was performed. This additional measurement (for the TLA treatment only) included measurements of air flow characteristics in seven additional measurement points (Table 1). Two measurement points were located outside of the BZ and the clean air-conditioned zone of the TLA (0.3m and -0.3m from the manikin’s nose in y-axe direction) and one point in the center of the horizontal plane (X = 0 and Y = 0) at a height of 0.23 m above manikin’s nose tip. The sampling locations were selected in accordance with the definition of BZ by Occupational Safety and Health Administration (OSHA) defining the breathing zone as an area within a 10-inch (0.25 m) radius of the people’s nose and mouth. The location of these measurement points together with overall experimental design for the ACS is summarized in Table 1.

**Table 1:** Location of anemometer sensors for the ACS for three tested ventilation settings.

Ventilation setting		Sensor position, m		
		X	Y	Z
**A**	**Without air cleaner**	0	0	0.08
**B**	**With room air cleaner**	0	0	0.08
**C**	**With TLA device**	0	0	0.08
**C**	With TLA device*	0	-0.10	0.08
**C**	With TLA device*	0	-0.20	0.08
**C**	With TLA device*	0	-0.30**	0.08
**C**	With TLA device*	0	0.10	0.08
**C**	With TLA device*	0	0.20	0.08
**C**	With TLA device*	0	0.30**	0.08
**C**	With TLA device*	0	0	0.23

*Investigation of airflow characteristics for ventilation setting C at seven additional locations.

**The sampling locations at Y = -0.3 m, Y = 0.3 were considered as measuring points out of the BZ.

### Chamber configuration and instrumentation

The experiments were performed in the 86 m^3^ environmental test chamber located at the Engineering Centre Bygholm in Horsens, Denmark. To minimize possible impact of heat loss, all walls, floor and ceiling were insulated with styrene-foam panels covered by steel plates. Fig 1 shows an experimental setup for both studies. Throughout the experiments the chamber was firmly closed and no outer air was supplied into the chamber.

The impact of a room air cleaner (ventilation setting B) was tested by use of a portable air cleaner (DA-5018E Honeywell, A/S) which was placed 1.2 m from the edge of the experimental bed (Fig 1). The air cleaner was equipped with a high efficiency particle arresting (HEPA) filter and provided constant volumetric air flow of 210 m^3^.h^-1^. The air provided by the air cleaner was not further conditioned. The exhaust of the air cleaner was at a height of 0.1 m above the floor and was directed towards the experimental bed.

**Fig 2:**
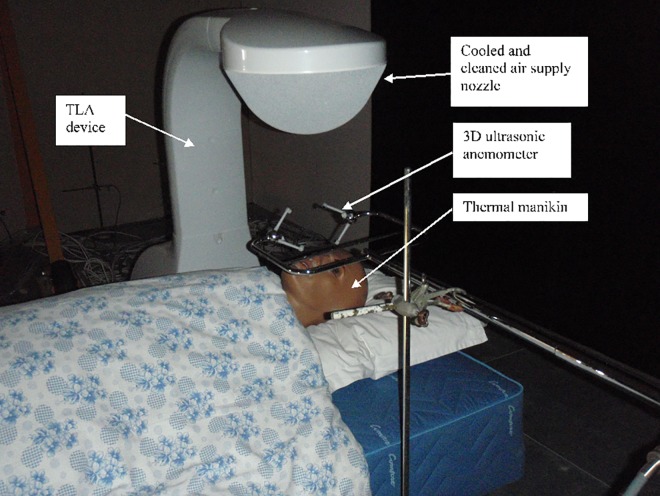
Experimental setup of the TLA device.

For ventilation settings C the TLA device (Airsonett AB, Sweden) was located on the right side of the bed with the supply nozzle above the manikin’s head (Fig 2). The manikin´s nose was considered as an initial origin of the three-dimensional positioning system (i.e. 0,0,0 [X,Y,Z]). The room air was sucked into the apparatus through an air intake located at the bottom of the TLA device. The air flow was further divided in two, of which one was cleaned through a HEPA filter and then was redirected into the integrated cooling system of the TLA device. The air was then distributed through a spherical-shaped nozzle with an opening diameter 0.46 m located 0.7 m above the tip of the manikin´s nose. The temperature of the supply air was approximately 1°C lower than the temperature of the ambient air. The air flow rate of the filtered air was estimated on the basis of measured values of air velocity and an opening area of the supply nozzle. The calculated air flow of filtered and cooled air was 125 m^3^. h^-1^. The second air flow was heated, filtered and blown through the air outlet at the back side of the apparatus (Fig 2).

The air flow characteristics in the BZ were monitored using a 3D ultrasonic anemometer (HS-50, Gill Instruments Ltd, UK), which permitted measurements with 2 Hz of sampling frequency. The anemometer measures the orthogonal components of the velocity vectors (U_x_, U_y_, U_z_; m.s^-1^) in the measured volume. The sensor is composed of three pairs of transducers providing measurement axes. The transducers are arranged in a manner that the velocity measurement represents a measuring volume of 3.75 x 10^−4^ m^3^ (0.15×0.05×0.05 m). The sensor was placed so that the longest axis of the measuring volume was positioned along the manikin’s face (see Fig 2). In total, the measuring events consisted of an effective measuring duration of 512 s, during which 1024 measurements were measured and evaluated.

The PCS included two sets of testing. The first set was designed to simulate a non-movement period of sleep. The entire measurement period lasted for 9 hours and included only one artificial elevation of PM–a duvet movement in the beginning of the period (t = 0 h). The concentrations of PM in the BZ were measured for all three ventilation settings. The second set of measurements was designed to represent a period with frequent moves in bed commonly performed by occupants before falls asleep [26]. Again the PM concentrations were artificially elevated by mechanical movements (liftings) of the duvet. The measured duration was 3 hours during which the duvet was lifted 13 times in 13 min and 48 sec intervals. We expected no impact of ventilation setting A on this set of measurement. Therefore only ventilation setting B and ventilation setting C were investigated.

To measure airborne particle number concentrations (#.cm^-3^) we used a laser-type Climet CI-500 Particle counter. The particle counter set consisted of the particle counter and a sampling tube with the end of the sampling tube located in the BZ of the thermal manikin, specifically over the point between the upper lip and the distal part of the nose. The particle concentrations were measured and divided into six particle-size bins: 0.3–0.5, 0.5–1.0, 1.0–5.0, 5.0–10, 10–25 and >25 μm. For further analyses the particles were grouped into two size fractions: “Fraction 1” included particles with diameter 0.3 to 0.5 μm and particles with aerodynamic diameter > 0.5 μm were grouped in “Fraction 2.” The grouping into two size fractions was decided in regards to the similar particle number concentrations in both groups.

### Experimental bed and thermal manikin

The thermal manikin was placed on a coil-type mattress (2 × 0.9 × 0.4 m) in the supine position. The experimental bed was further equipped with a pillow covered by a cotton-type pillow cover. A cotton-type duvet with polyester filling was covered by a cotton-type, high-treated count duvet cover.

The manikin was built of fiber glass and originally was used for clothing display. The manikin represented an average adult person with height 1.75 m. A heating cable was placed inside the head to simulate the thermal convection of air due to the heat transfer from the surface of the manikin´s head. The temperature of the heating cable was adjusted by changing the voltage over the cable to obtain a human-like surface temperature and its distribution, which was controlled by comparing the thermal pictures of human and manikin faces taken by a thermal- vision video camera (NEC TH 7102). For the PCS an additional heat plate was placed between the thermal manikin and mattress to simulate the heat production generated by a human body.

### Parameters for airflow characteristics and motion of airborne particle dynamics

The three-dimensional resultant air velocity (U; m.s^-1^) in the measuring volume was calculated using:

$$U=\sqrt{U_{X}^{2}+U_{Y}^{2}+U_{Z}^{2}}$$ (1)

Where U_X_, U_Y_ and U_Z_ are velocities in X, Y and Z direction. The U was used to calculate Reynolds Number. The Reynolds number (Re, dimensionless) is a ratio between kinetic force and viscos force in a flow and indicates the relative importance of the forces.

Re=ρUDμ (2)

where ρ is the density of the air (in the present study: 1.293 kg.m^-3^), μ is the dynamic viscosity of the air provided by the TLA (in the present study: 18×10^−6^ N.s.m^-2^) and D (m) is a characteristic dimensional scale. The characteristic dimensional scale was set as 0.2 m which is equal to the width of the manikin´s head. Reynolds number for a particle (Re_p_) is given by:

Rep=dvρμ (3)

where d is the aerodynamic particle diameter (m) and v is the air velocity relative to the particle (m.s^-1^), i.e. the terminal velocity of the particle.

The three-dimensional turbulence intensity (TI, %) expresses the magnitude of the time-averaged velocity fluctuations of the turbulent motions as a proportion of the mean velocity, i.e. instability of the airflow. The TI can be expressed as:

$$\mathrm{TI}=100\frac{\sigma }{\bar{U}}$$ (4)

where *σ* is the standard deviation of *U* and Ū is the mean value of U (m.s^-1^). Describing Eq 4 for each direction, the turbulence intensity of wind vector components (TI_x_, TI_y_, TI_z_) were calculated by:

$${\mathrm{TI}}_{X}=100\frac{\sigma_{X}}{\bar{U}}$$ (5)

$${\mathrm{TI}}_{Y}=100\frac{\sigma_{Y}}{\bar{U}}$$ (6)

$${\mathrm{TI}}_{Z}=100\frac{\sigma_{Z}}{\bar{U}}$$ (7)

where σ_x_, σ_y_, σ_z_ are the standard deviation of wind vector components (m.s^-1^) for direction X, Y and Z respectively. The time required for an eddy to flow past a fixed position can be characterized by Eulerian integral time scale (T_i_, s), which is obtained by integrating the auto-correlation coefficient to the first zero crossing [27]. In this study, T_i_ can be defined as:

$$T_{i}=\sum_{k=1}^{k=j}\left(R_{k}\frac{1}{f_{s}}\right)$$ (8)

where R_k_ (dimensionless) is the Lagrangian autocorrelation coefficient and f_s_ is the measuring frequency (s^-1^). The index j refers to the first zero crossing, i.e negative R_k_ of the correlogram. The R_k_ can be defined as a division of a covariance (C_k_, m^2^.s^-2^) and squared value of σ. Therefore

$$R_{k}=\frac{C_{k}}{\sigma^{2}}$$ (9)

The covariance in Eq 9 represents the property of the turbulent flow:

$$C_{k}=\frac{1}{n}\sum_{i=1}^{i=n-k}\left\{\left(U_{i}-\bar{U}\right)\left(U_{i+k}-\bar{U}\right)\right\}$$ (10)

where n is the number of measurements, index k represents values measured in steps one to (n-1). Integral length scales (*L*_*i*_, m) is to a certain extent a measure of the longest airflow connection, or correlation distance, between the velocities at two positions of the flow [27]. *L*_*i*_ indicates the size of eddies of the maximum energy in the flow field and scale of mixing action. For the purpose of this study L_i_ can be define defined as:

$$L_{i}={\bar{U}T}_{i}$$ (11)

Turbulence kinetic energy (TKE, m^2^.s^-2^) is the mean kinetic energy per unit mass associated with eddies in turbulent flow

$$\mathrm{TKE}=\frac{1}{2}\times \left(\sigma_{X}{}^{2}+\sigma_{Y}{}^{2}+\sigma_{Z}{}^{2}\right)$$ (12)

Mixing in turbulent airflow (diffusivity can be described in terms of transport or diffusion of turbulent energy or turbulent momentum in the airflow), first-order approximation for diffusivity (D_if_, m^2^/s^2^) can be proposed as [28]:

$$D_{\mathrm{if}}=L_{i}\mathrm{RMS}\left(U\right)$$ (13)

This may be considered as a measure indicating the ability of the turbulent airflow to transport airborne particles relating to the distance referring to *L*_*i*_.

### Particle-removal effectiveness

Two metrics were used to estimate the overall effectiveness of the air cleaners: Reduction Effectiveness (H, %) and Cleaning Efficiency (CE, #.m^-3^h^-1^). Reduction Effectiveness H has been previously defined and introduced by [29]:

$$H=\frac{\left(C_{0}-C_{t}\right)}{C_{0}}\;∙\;100$$ (14)

where c_0_ is the airborne particle number concentration at the beginning and c_t_ is the airborne particle number concentration in time t (at the end of the measured cycle). Cleaning efficiency (CE) evaluates a total number of airborne particles removed per unit of time:

$$CE=\frac{\left(C_{0}-C_{t}\right)}{\left(t_{t}-t_{0}\right)}$$ (15)

where the nominator of the fraction in Eq 15 represents elapsed time from the beginning of the measured period (t_0_).

### Software

Microsoft Office® Excel ver. 2010 was used for data analyses and graphical presentations. For the vector presentation open source graph drawing software Graph R version 2.32 was used.

## Results and Discussion

### Airflow characteristics

**Fig 3:**
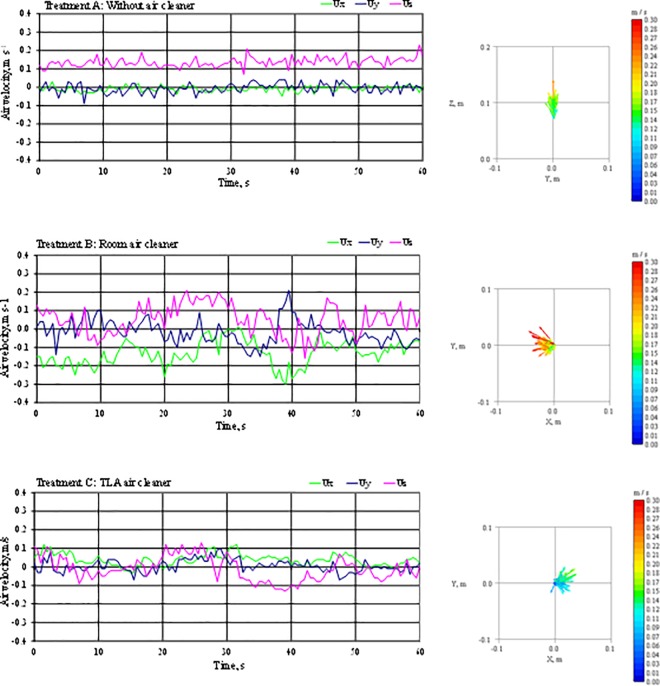
Results from 3D air velocity measurements at 8 cm above the nose tip. The results are presented in terms of orthogonal air velocity components (Ux, Uy, Uz) and the airflow.

This section presents the impact of different ventilation techniques on airflow in the BZ of a sleeping person. Fig 3 shows the results from air flow measurements over a period of 60 seconds for three ventilation settings: without any ventilation operating (Fig 3A), with a room air cleaner operating (Fig 3B) and under TLA treatment (Fig 3C). During all three settings, the velocity probe was located 8 cm above the tip of the manikin´s nose. In Fig 3A, 3B and 3C the U_X_ and U_Y_ are horizontally-oriented air velocity vectors and U_Z_ is a vertical air velocity vector. The velocity components are assessed as air velocity for each direction and as a three-dimensional resultant air velocity vector U. The average air velocity ranged from 0.09 m.s^-1^ for the TLA air cleaner to 0.17 m.s^-1^ for the room air cleaner (Table 2).

**Table 2:** Results from airflow characteristics study for tested ventilation settings A, B and C.

		Setting A: Without air cleaner	Setting B: Room air cleaner	Setting C: TLA device
**Sensor position, m**	**X**	0	0	0
	**Y**	0	0	0
	**Z**	0.08	0.08	0.08
**Average air velocity (Ū), m/s**	**Ū**	0.14	0.17	0.09
	**Ū**_**X**_	0.00	-0.10	0.05
	**Ū**_**Y**_	0.01	-0.01	0.00
	**Ū**_**Z**_	0.13	0.05	0.02
**Turbulence intensity (TI), %**	**TI**	18	32	39
	**TI**_**X**_	11	40	34
	**TI**_**Y**_	19	50	35
	**TI**_**Z**_	19	53	77
**Reynodls number for D = 0.2 m**		1,983	2,480	1,269
**Time scale (T**_**i**_**), s**		0.1	1.0	1.6
**Length scale (L**_**i**_**), m**		0.01	0.18	0.14
**Turbulence diffusivity (Dif), m**^**2**^**/ s**		1.2E-03	3.3E-02	1.4E-02
**Turbulence kinetic energy (TKE), m**^**2**^**/s**^**2**^		7.9E-04	1.0E-02	3.3E-03

Ventilation setting A showed the smallest velocity fluctuations in all three directions compared to the other ventilation settings. The horizontally-oriented velocity components U_X_ and U_Y_ were close to 0 m.s^-1^ and the vertical velocity U_Z_ ranged from 0.08 m.s^-1^ to 0.22 m.s^-1^ with average 0.13 m.s^-1^. Because no ventilation was operating the only force influencing the air flow was the thermal plum. The vertical heat flux generated by the thermal plum created mean velocities in the direction identical to free convective flow velocity directions reported in previous studies [30, 31, 32] reaching up to 0.25 m.s^-1^ for the head region. Velocities reported in these studies were up to two-time higher compared to our study. However, these velocities have not been measured for laying person as in our study, but for seated or standing person where the air originates at foot level and the velocity grew as the air moves towards the head region. Also, the duvet served as a barrier for heat flux generated by the rest of the body under the duvet and the only active thermal plum-generated part of the manikin was the manikin’s head.

The same studies found the thickness of the free convective flow in the head area to be approximately 0.2 m for fully developed free convection flow. Looking at measured results presented in Table 2 we can describe the parameters of the thermal plum of our tested manikin: the velocity U_Z_ was the highest among all three settings (0.13 m.s^-1^), close-to-zero values of U_X_ and U_Y_, and Reynolds number in a range for laminar air flow (Re = 1983). The turbulence intensity (TI) during this setting was lowest of all three settings either in each individual direction (11, 19 and 19%) as well as in overall 3-dimensional value (TI <19%). This setting also showed lowest turbulence kinetic intensity (TKE = 7.9 x 10^−4^ m.s^-2^), turbulence diffusivity (D_if_ = 1.2 10^−3^ m^2^.s^-1^), Length scale (L_i_ = 0.01 m) and Time scale (T_i_ = 0.1). The TKE, D_if_, Li and T_i_ were usually one to two orders of magnitude lower compared to the results obtained when air movement in the breathing zone was present, i.e. during ventilation settings B and C. These results indicate that the thermal plum generated only by the manikin’s head is thicker than 0.08 m and is characterized by an upward laminar free convection flow with lower upward velocities compared to the fully developed free convection flow of a standing person.

The ventilation setting B showed the highest velocity fluctuation in the horizontal plane. The effect of the room air cleaner can be observed also from the measured average air velocity (U = 0.17 m.s^-1^), the highest of all three ventilation settings. The strength of the air momentum generated from the room air cleaner is also demonstrated by the U_X_ being two-times higher than the measured U_Z_. In other words, the interaction between the free convection flow (in vertical direction) and the air flow from the room air cleaner (in horizontal direction) resulted in overpowering of the vertical free convection flow by the oncoming air flow from the room cleaner. The interaction between a free convection flow and an oncoming air flow has been studied previously. [13] studied air flow from small jets as a personal ventilation method and showed that oncoming air flow must have a certain momentum and velocity to penetrate the BZ and deliver clean air. [13] also studied minimal velocities needed to effectively penetrate the personal convection flow of a standing person. According to his measurements, 0.3 m.s^-1^ is a velocity high enough to penetrate completely the free convection flow around human head and deliver 100% clean air. However, our measurements with no source of disruptions (ventilation setting A) showed that the free convective flow was not yet fully developed as in the case of a standing person. Thus lower velocity would be needed to penetrate the personal flow which based on the velocity result, happened with the room air cleaner operating. This setting was associated with highest TKE (10^−2^ m^2^.s^-2^), L_i_ (0.18 m), D_if_ (3.3 x 10^−2^ m^2^.s^-1^) and Reynolds number (Re = 2480) of the three investigated ventilation settings. High turbulence intensities in the horizontal plane TI_X_ and TI_Y_ and the highest turbulence diffusivity D_if_ of all tested settings indicate that the filtered air prior to approaching the BZ probably mixed with surrounding air and therefore increased the pollutant concentration in the BZ. Further evaluation of the impact of the room air cleaner is described in chapter 3.3. Surprisingly, the velocity vector U_X_ was not directed in the room air cleaner-experimental bed direction, but in the opposite direction, i.e. towards the room air cleaner as a result of a return flow from the wall S. This is most likely due to the air distribution in the room. The air stream was blown in an acute angle (approximately 40° from horizontal plane) and it was the return air flow that entered the BZ.

In summary, the high turbulence intensity, high velocity and turbulence diffusivity level indicate weak effect on the clean air delivery performance and possible disturbance of a sleeping person by a draft. Also, the geometry of the room and the relative placement of the room air cleaner influences strongly the air flow characteristics in the BZ when room air cleaner was operating.

The TLA treatment showed the smallest upward average velocity U_Z_ = 0.02 m.s^-1^. However, Fig 3C shows high velocity fluctuation to both negative and positive values. TI_Z_ for the TLA treatment was 77%, which is much larger than all other observed turbulence intensities (Table 2). This indicates that the two opposite direction airflows, i.e. downward airflow from the TLA air supply nozzle and upward plume from the manikin, met and couldn’t establish a balance, which resulted in fluctuating air movements. However, for the TLA device the turbulence diffusivity was one fold lower compared to when no ventilation was operating and the Reynolds number was the lowest of all three settings (Re = 1269). This is explainable by the parameters of the air supply distribution. In particular, slightly lower temperature and laminar air flow of the supply air penetrate more effectively the upward plume from sleeping person and deliver larger amounts of clean air into the BZ. Additionally, length scales (L_i_) of the settings B and C were at the same level and both were at comparable sizes as manikin’s face. This means that the transportation distances of airborne particles for both settings should be about the same, i.e. the particles raised around the face are expected to be transported at a comparable distance as the manikin face by the vortex.

Overall, the measurements showed that in a case where no air cleaner is operating the main driving force of the air flow in the BZ is the free convective flow generated by the temperature difference between the surface temperature of the manikin and the room air temperature. The room air cleaner can deliver a high momentum turbulent air flow into the BZ and overpowers the free convective flow entirely. Opposite from the room air cleaner the TLA device creates laminar air flow with opposite direction than the free convective flow and penetrates the BZ without creating excessive drafts.

### Airflow characteristics in the cooled and cleaned airflow of the TLA

This sub-study focuses on further measurements of air flow characteristics for the TLA device. The air flow characteristics were measured in seven points along the y-axis of the coordination system (Table 3).

**Table 3:** Airflow characteristics in the cleaned and cooled air zone created by TLA device.

**Sensor position**	**X**	0	0	0	0	0	0	0	0
	**Y**	-0.30	-0.20	-0.10	0	0.10	0.20	0.30	0
	**Z**	0.08	0.08	0.08	0.08	0.08	0.08	0.08	0.23
**Average sir velocity (Ū), m/ s**	**Ū**	0.17	0.12	0.10	0.09	0.09	0.11	0.10	0.15
	**Ū**_**X**_	0.02	0.00	0.02	0.05	0.03	0.01	0.01	-0.01
	**Ū**_**Y**_	-0.04	-0.04	-0.04	0.00	0.02	0.03	0.02	0.01
	**Ū**_**Z**_	-0.16	-0.11	-0.07	0.02	-0.07	-0.11	-0.10	-0.15
**Turbulence intensity (TI), %**	**TI**	18	22	28	39	30	18	33	7
	**TI**_**X**_	9	12	26	34	30	15	14	4
	**TI**_**Y**_	19	20	31	35	33	14	22	6
	**TI**_**Z**_	18	24	45	77	44	22	31	7
**Rynolds number for D = 0.2m (Re)**		2427	1765	1377	1269	1304	1620	1491	2,121
**Time scale (T**_**i**_**), s**		0.4	2.2	0.6	1.6	1.0	0.7	3.0	8.0
**Length scale (L**_**i**_**), m**		0.07	0.27	0.05	0.14	0.09	0.08	0.32	1.18
**Turb. Diffusivity (Dif), m**^**2**^**/s**		1.1E-2	3.5E-2	5.4E-3	1.4E-2	8.9E-3	9.2E-3	3.5E-2	1.7E-1
**Turb. kinetic energy (TKE), m**^**2**^**/s**^**2**^		1.1E-3	8.4E-4	1.7E-3	3.3E-3	1.6E-3	5.8E-4	8.9E-4	1.1E-4

The average air velocity U ranged from 0.09 m.s^-1^ in the center of the horizontal plane [0, 0, 0.08] and increased with increasing distance from the center of the origin to both sides. However, the calculated U describes only the size of the velocity vector, not its direction. To describe the air flow characteristics in more detail the velocity vectors must be assessed individually in each direction, i.e. U_X_, U_Y_, and U_Z_. The vertical velocity vector U_Z_ has negative values (downward flow) in all measurement points except in the center where the velocity vector changes to an upward direction. To better describe the airflows interaction in the head region it is important to know convective flow patterns around the head of a sleeping person. Ideally, with no impact of thermal manikin the TLA device would create laminar downward air flow. [30] investigated convective flow patterns around the human head in the supine position. They found that the free convective flow thickens only one centimeter on both sides of the head when approximately at the forehead level and the plume tapers off symmetrically as the flow rises above the head. The tapering effect of the warm air stream helps to form the flow and gives it higher momentum. The higher momentum of the upward air flow and the downward flow from the TLA device reached equally strong upward lift from the convective flow. Further from the center the upward free convective flow weakened and was overtaken by the downward flow from the TLA device as indicated by increasing values of U_Z_ in downward direction (Table 3).

Further support of this observation can be drawn by assessing the turbulence intensity. The turbulence intensity increased as it got closer to the center indicating that the two airflows created unstable environment characterized by fluctuating air movement. Despite relatively high turbulence intensity the air flow was laminar with Re ranging from 1377 to 1765 except in the measurement point far left (0, -0.3, 0.08) where the Re was in lower end of transient flow range.

**Fig 4:**
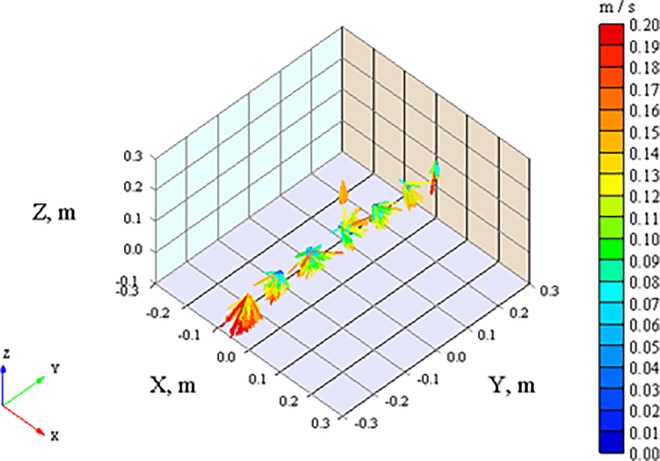
The airflow velocity vectors determined under the TLA cooled air supply nozzle. Displayed are measured positions: 23 cm above the nose tip (X = 0, Y = 0, Z = 0.23m), 8 cm above the nose tip (X = 0, Y = 0, Z = 0.08m), right and left side of the face (X = 0, Y = ±0.2, Z = 0.08) and (X = 0, Y = ±0.3, Z = 0.08m).

Fig 4 displays 3-D scheme of measured velocity vectors. The horizontal-plane vectors U_X_ and U_Y_ are directed away from the center. This is most likely due to the downward stream enlarging the span to flow around the obstacle (head of the manikin). Although the velocity vectors of U_X_ are oriented toward the manikin’s forehead only, the values are close to zero (Table 3).

One additional measurement point was later included to measure air flow characteristic at height 0.23 m above the manikin’s nose [0, 0, 0.023]. The airflow beneath the TLA air supply in this measurement point with an average resultant air velocity was 0.15 m.s^-1^. The TKE of the outlet air was about 30 times smaller than the TKE above the nose tip (0, 0, 0.08). The TKE reduced quickly as the measuring point moved to the sides. The D_if_ of the outlet air was 12 times greater than the D_if_ above the nose tip. At this point, the horizontal-plane velocities were close to zero while absolute value of U was equal to absolute value of U_Z_ with velocity vector facing downward. This means that at the height of 0.23m the downward laminar flow from the TLA device is undoubtedly dominating encompassing the BZ.

### Particle concentrations in the breathing zone

The following section presents the evaluation of particle-removal performance during periods with and without periodic particle-generating movements. The particle-removal performances were evaluated using reduction rate (Eq 14) and cleaning efficiency (Eq 15). Both measurements represent a situation of a person in a bed in the supine position. The non-movement period of 9 hours simulated sleep period during which a sleeping person creates minor movements mainly with limbs or head [33]. The second set of measurement with periodic duvet lifting simulated the impact of movements while people sleep previously reported by [34] and [35]. This period is characterized by frequent moves, such as torso turns, changing position from supine to prone etc. [34] reported position shifts in different age groups ranging from 4.7 position shifts per hour in 8–12 year old children to 2.1 in 65–80 year old adults. In our study the performed simulation with duvet movement in this study were designed to be within the range of [34] data.

**Fig 5:**
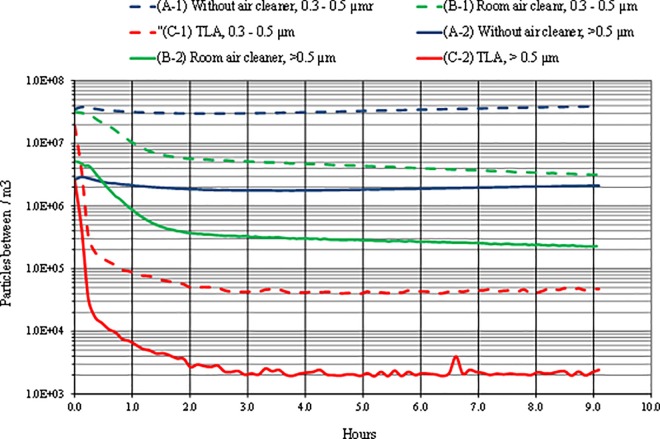
Time series variation of concentrations of two particle size fractions in the breathing zone with three different treatments applied. A) without air cleaner, B) with room air cleaner and C) with TLA device. (Fraction 1 includes the particles between 0.3 and 0.5 μm. Fraction 2 includes the particles larger than 0.5 μm.)

Fig 5 shows particle concentrations for three ventilation settings, each for two particle-size fractions. The initial particle concentrations ranged from 2x10^6^ #.m^-3^ to 5x10^6^ #.m^-3^ for Fraction 2 and 2x10^7^ #.m^-3^ to approximately 4x10^7^ #.m^-3^ for Fraction 1.

**Fig 6:**
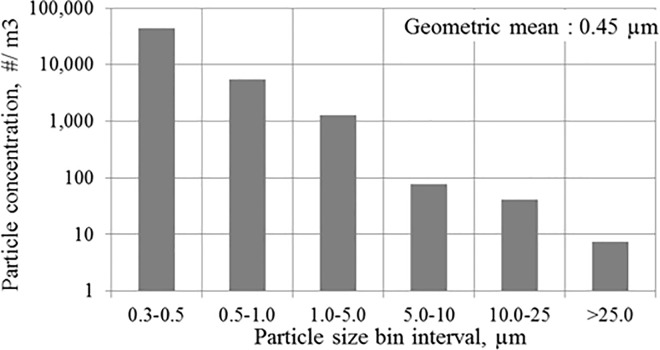
Overall particle distribution over the entire measurement period with periodic movements. Measuring interval: 23 s, i.e. 479 measurements for each particle size bin interval.

In a situation with no air filtration the concentration of Fraction 1 decreased insignificantly and in the second half of the measured period the concentration increased slightly. This result is expected due to the lower deposition rate typical for small particles. It has been shown by sleep environment studies that smaller particles tend to stay airborne prolonged period before settling down back down on the bedding surface [11, 12]. [11] and [12] found in their experiments that during a period without any movement the only particle-removal was caused by the main HVAC system in the experimental chamber. A similar trend can be observed in our study: small particles tend to stay airborne and with the lack of any air filtration the particle concentration stays almost constant. This finding is also in accordance with previous studies focusing on particle behavior elsewhere in the indoor environment [36, 37] which has very important meaning. Longer periods during which smaller particles stay airborne increase the likelihood of being inhaled rather than being removed from the BZ via gravitational settling to the mattress surface (i.e. settle down). Surprisingly the concentration reduction for Fraction 2 in our study for which one may assume that the gravitational mechanism will help to decrease the concentration level, was not significant either (Fig 5). The explanation might be drawn from the particle-size distribution in the fraction 2 as shown in Fig 6. Fig 6 shows overall particle distribution over the entire measurement with periodic movements. The largest portion of Fraction 2 consisted from particles in the lower end of the size range characterized by lower deposition rate. The deposition of large particles included in the Fraction 2 had minimal effect on the total particle number.

When the room air cleaner was operating the particle concentration decay profile for Fraction 1 is almost identical as for Fraction 2. The only measured difference between Fraction 2 and Fraction 1 were the initial concentrations, higher for Fraction 1. Table 4 presents reduction rates for the room air cleaner and the TLA device. The room air cleaner shows significantly lower reduction rates compared to the TLA device. In other words, the room air cleaner needs more time to remove the same portion of particles in BZ than the TLA. While the TLA device removes over 80% of all small airborne particles in less than 8 minutes (0.118 h), the room air cleaner reaches the similar removal rate in 2 hours.

**Table 4:** Effectiveness of room air cleaner and TLA air cleaner.

**Duration of the operation, h**	**Setting B: Room air cleaner**		**Setting C: TLA device**	
	**Fraction 1**	**Fraction 2**	**Fraction 1**	**Fraction 2**
	**Reduction rate (H), %**			
0.118	3.0	7.1	80.3	82.5
0.5	32.3	51.5	99.2	99.5
1.5	79.1	90.8	99.7	99.8
2.0	82.1	92.8	99.7	99.9
9.0	89.9	95.5	99.7	99.9
	**Cleaning efficiency (CE) (#/m**^**3**^ **h**^**1**^**)**			
0.118	8.1E+06	3.1E+06	1.3E+08	1.8E+07
0.5	2.2E+07	5.6E+06	4.0E+07	5.3E+06
1.5	1.7E+07	3.2E+06	1.2E+07	1.7E+06
2.0	1.3E+07	2.4E+06	9.5E+06	1.3E+06
9.0	3.2E+06	5.4E+05	2.1E+06	2.8E+05

The removal rate for larger particles (Fraction 2) is higher than for smaller particles, however, still far below the removal rate of the TLA device. The differences between the results of the two air cleaners are due to the differences in the air cleaning principles. While the TLA cleanse relatively small volume encompassing manikin’s head and the BZ, the room air cleaner mixes the filtered air with “polluted” air in the chamber. Instead of cleaning small volume the room cleaner must clean the entire air volume of the experimental chamber due to the air-mixing effect. This results in delivering of air partly filtered and partly mixed with ambient polluted air into the BZ and thus negatively influenced the particle-removal performance of the room air cleaner. The use of the TLA device showed the highest reduction rate for both fractions showed which was capable of removing over 99% within 30 minutes. Similar values of reduction rate in the rest of the measurement period means the TLA device reaches low concentration (99.9% of initial particles removed) within a short period of time and is able to protect the BZ from further transport of particles from peripheral zone. Similar results were observed by [22] comparing real-person exposure to small and large positioned in bed under two conditions: the TLA device operating and not operating.

The TLA device showed higher cleaning efficiency for all calculated time points for both fractions when compared to cleaning efficiency of the room air cleaner. It is due to the different amounts of clean air delivery. The TLA device delivers cooler dense air which creates a volume encompassing the BZ with low mixing with air from peripheral zone. Considering this small volume and recirculation rate 125 m^3^.h^-1^, the volumetric air exchange rate is far higher than the volumetric air exchange rate for the room air cleaner (replacing the entire experimental chamber volume).

**Fig 7:**
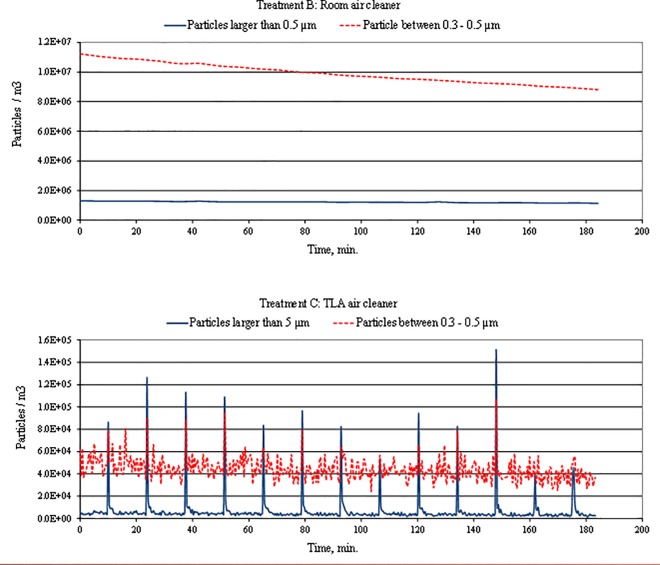
Variations of particle concentrations in the breathing zone when air cleaner (setting B) and TLA device (setting C) was used and duvet was lifted periodically.

This difference can be observed from concentration measurements with periodic duvet movements. The duvet movement has no effect on the concentration profile for either Fraction 1 or Fraction 2 (Fig 7A). The air mixing effect caused by the room air cleaner levels the particle concentration in the BZ with particle concentration in the experimental chamber. The room air cleaner behaves as a displacement or centralized HVAC system, which has been shown as ineffective in delivering clean air into the BZ. With the TLA treatment, there were reductions of similar magnitude to study of [22]. As in [22] study our measurement showed a rapid decrease of large particles in the breathing zone almost immediately after each duvet movement. On the other hand, reduction of small particles was less efficient than for large particles. This could be explained by a low deposition rate of small particles. Another contributory factor may be the fact that end tip of the sampling tube was located the upper lip and the distal part of the nose. In this area the thermal plum from the manikin’s head had a much stronger effect on small particle behavior than the TLA device. The number of particles of Fraction 1 is normally 5 to 10 times greater compared to Fraction 2 in the ambient room air which is confirmed by the comparison of the fractions without air cleaner (Fig 6). Hence, the filtered supply air from both the TLA device and the room air cleaner will contain at least same ration. Furthermore, the filter removal efficiency is lower for smaller particles which may increase the concentration ratio. Another source might be a possible intrusion of particles from ambient air which also will contain 5–10 times more particles of Fraction 1. Also, we hypothesize that the heating plate placed between the manikin and the mattress might have caused air movement under the duvet associated with particle transport from below the duvet to the BZ through small openings. Such air movement can transport small particles from under the duvet, but might have not enough momentum to transport larger particles. The particle concentration fluctuated between 4x10^4^ to 6x10^4^ #.m^-3^, similarly to measurement with no movements. This indicates that for small particles the number of movements in the bed has negligible impact on particle concentration profile, similarly to findings in previous studies.

This paper reports only particle-removal effectiveness of selected air removal techniques in a laboratory environment. The measurements did not include the impact of the breathing process which may significantly influence the concentration profile and level of mixing with air from peripheral zones under TLA treatment. Furthermore, the investigation only simulated a real human body and focused solely on measurement in the supine position. The impact of different positions and breathing processes on particle-removal efficiencies of TLA treatment is the subject of a follow-up study.

## Conclusion

Full-scale chamber experiments were conducted to investigate the effect of controlled laminar air flow on particle number concentration in the breathing zone. The results were compared to situations when a portable room air cleaner was operating and when no ventilation system was used. Based on the objectives stated in the introduction chapter three main conclusion can be drawn:

1. A laying person (under duvet) generates laminar free convection flows similar to standing or sitting person with lower air flow velocities.
2. Room air cleaner has a tendency to create strong drafts overpowering the free convective flow and with higher mixing rate with outer air. This leads to lower particle-removal performance in the breathing zone of the sleeping person.
3. The TLA device is capable of reducing particle concentrations of particles almost instantly despite the number and intensity of particle-resuspending movements. The TLA device provides a barrier which significantly reduces the intrusion of airborne particles into the breathing zone with particle number concentrations of a 100-fold lower compared to an room air cleaner.

Further studies should be conducted for the understanding of the transport of resuspended particles between the duvet and the laying body

## References

[1] Bin Y. S., Marshall N. S., and Glozier N. Secular trends in adult sleep duration: a systematic review. Sleep medicine reviews. 2012; 16(3), 223–230. 10.1016/j.smrv.2011.07.003 22075214

[2] Klepeis Neil E., Nelson William C., Ott Wayne R., Robinson John P., et al The National Human Activity Pattern Survey (NHAPS): a resource for assessing exposure to environmental pollutants. Journal of exposure analysis and environmental epidemiology. 2001; 11, 231–252. 10.1038/sj.jea.7500165 11477521

[3] Custovic A., Taggart S.C., Francis H.C., Chapman M.D. and Woodcock A., 1996. Exposure to house dust mite allergens and the clinical activity of asthma. Journal of Allergy and Clinical Immunology. 1996; 98(1), pp.64–72. 8765819 10.1016/s0091-6749(96)70227-0

[4] Ross R.N., Nelson H.S. and Finegold I. Effectiveness of specific immunotherapy in the treatment of asthma: a meta-analysis of prospective, randomized, double-blind, placebo-controlled studies. Clinical therapeutics. 2000; 22(3), pp.329–341. 10.1016/S0149-2918(00)80037-5 10963287

[5] IOM (Committee on the Assessment of Asthma and Indoor Air of the Institute of Medicine). 2000 Clearing the Air: Asthma and Indoor Air Exposures. Washington, DC:National Academies Press.

[6] Kanchongkittiphon W, Mendell MJ, Gaffin JM, Wang G, and Phipatanakul W. Indoor environmental exposures and exacerbation of asthma: an update to the 2000 review by the Institute of Medicine. Environ Health Perspect. 2015; 123:6–20 10.1289/ehp.1307922 25303775 PMC4286274

[7] Murray C.S., Poletti G., Kebadze T., Morris J., Woodcock A., Johnston S.L. et al Study of modifiable risk factors for asthma exacerbations: virus infection and allergen exposure increase the risk of asthma hospital admissions in children. 2006 pp.376–382. 10.1136/thx.2005.042523 16384881 PMC2111190

[8] Dorward A. J., Colloff M. J., MacKay N. S., McSharry C., and Thomson N. C.. Effect of house dust mite avoidance measures on adult atopic asthma. 1988 Pp.98–105. 3353895 10.1136/thx.43.2.98 PMC1020749

[9] Platts-Mills T., Tovey E. R., Mitchell E. B., Moszoro H., Nock P., and Wilkins S. R.. Reduction of bronchial hyperreactivity during prolonged allergen avoidance. Lancet. 1982; 2:675–678. 6126624 10.1016/s0140-6736(82)90709-7

[10] Warner J. O., and Boner A. L.. Allergy and childhood asthma In: Clinical Immunology and Allergy, Kay A. B., ed. London, England: Bailliere Tindall 1988 pp. 217–224.

[11] Boor B. E., Spilak M. P., Corsi R. L. and Novoselac A. Characterizing particle resuspension from mattresses: chamber study. Indoor Air 2015; 25: 441–456. 10.1111/ina.12148 25077669

[12] Spilak M. P., Boor B. E., Novoselac A., and Corsi R. L. Impact of bedding arrangements, pillows, and blankets on particle resuspension in the sleep microenvironment. Building and Environment. 2014; 81, 60–68.

[13] Bolashikov Z., Melikov A., Spilak M., Nastase I. and Meslem A. Improved inhaled air quality at reduced ventilation rate by control of airflow interaction at the breathing zone with lobed jets. HVAC&R Research. 2014; 20(2), 238–250.

[14] Laverge J., Spilak M. and Novoselac A. Experimental assessment of the inhalation zone of standing, sitting and sleeping persons. Building and Environment. 2014; 82, pp.258–266.

[15] Bolashikov Z., Melikov A. and Spilak M. Experimental investigation on reduced exposure to pollutants indoors by applying wearable personalized ventilation. HVAC&R Research. 2013; 19(4), 385–399.

[16] Rim D. and Novoselac A. Transport of particulate and gaseous pollutants in the vicinity of a human body. Building and Environment. 2009; 44(9), 1840–1849.

[17] Rim D., and Novoselac A. Occupational exposure to hazardous airborne pollutants: Effects of air mixing and source location. Journal of occupational and environmental hygiene. 2010; 7(12), 683–692. 10.1080/15459624.2010.526894 20981607

[18] Bräuner E. V., Forchhammer L., Møller P., Barregard L., Gunnarsen L., Afshari A., et al Indoor particles affect vascular function in the aged: an air filtration–based intervention study. American Journal of Respiratory and Critical Care Medicine. 2008; 177(4), 419–425. 10.1164/rccm.200704-632OC 17932377

[19] Habbick B.F., Pizzichini M.M., Taylor B., Rennie D., Senthilselvan A. and Sears M.R. Prevalence of asthma, rhinitis and eczema among children in 2 Canadian cities: the International Study of Asthma and Allergies in Childhood. Canadian Medical Association Journal. 1999; 29;160(13):1824–8. 10405666 PMC1230435

[20] Goetzsche PC and Johansen HK. House dust mite control measures for asthma. Cochrane Database Syst Rev. 2008; 10.1002/14651858.CD001187.pub3 PMC8786269 18425868

[21] Kilburn S, Lasserson TJ and McKean MP. Pet allergen control measures for allergic asthma in children and adults. Cochrane Database Syst Rev. 2003; 10.1002/14651858.CD002989 PMC8689577 12535446

[22] Gore R.B., Boyle R.J., Gore C., Custovic A., Hanna H., Svensson, et al Effect of a novel temperature‐controlled laminar airflow device on personal breathing zone aeroallergen exposure. Indoor air. 2015; 25(1), pp.36–44. 10.1111/ina.12122 24750266

[23] Pedroletti C., Millinger E., Dahlen B., Söderman P. and Zetterström O. Clinical effects of purified air administered to the breathing zone in allergic asthma: a double-blind randomized cross-over trial. Respiratory medicine. 2009; 103(9), pp.1313–1319. 10.1016/j.rmed.2009.03.020 19443189

[24] Boyle R.J., Pedroletti C., Wickman M., Bjermer M., Valovirta E., Dahl R., et al Nocturnal temperature controlled laminar airflow for treating atopic asthma: a randomized controlled trial. Asthma and the environment. 2012; 67, 215–221. 10.1136/thoraxjnl-2011-200665 PMC3282042 22131290

[25] Schauer U., Bergmann K.C., Gerstlauer M., Lehmann S., Gappa M., Brenneken, et al Improved asthma control in patients with severe, persistent allergic asthma after 12 months of nightly temperature-controlled laminar airflow: an observational study with retrospective comparisons. European Clinical Respiratory Journal. 2015; 10.3402/ecrj.v2.28531 PMC4629753 26557252

[26] Aaronson S.T., Rashed S., Biber M.P. and Hobson J.A. Brain state and body position: a time-lapse video study of sleep. Archives of General Psychiatry. 1982; 39(3), pp.330–335. 7065843 10.1001/archpsyc.1982.04290030062011

[27] Hinze J. O. Turbulence. McGraw-Hill New York, 1975.

[28] Vincent James H. Aerosol Sampling–Science and practice, Inst. Of Occupational Medicine, Edinburgh, UK, John Wiley & Sons 1989; ISBN 0 471 92175 0: 390 p.

[29] Miller-Leiden S., Lohascio C., Nazaroff W. W., and Macher J. M. Effectiveness of in-room air filtration and dilution ventilation for tuberculosis infection control. Journal of the Air & Waste Management Association. 1996; 46(9), 869–882. 8806221 10.1080/10473289.1996.10467523

[30] Clark R. P., and Cox R. N. An application of aeronautical techniques to physiology 1. The human microenvironment and convective heat transfer. Medical and biological engineering. 1974; 12(3), 270–274. 4466977 10.1007/BF02477790

[31] Melikov, A. and Zhou, H. Comparison of methods for determining teq under welldefined conditions. In CABCLI seminar dissemination of results from EQUIV-project.1999; pp. 41–52.

[32] Özcan O., Meyer K.E. and Melikov A.K. A visual description of the convective flow field around the head of a human. Journal of Visualization. 2005; 8(1), pp.23–31.

[33] Lauderdale D.S., Knutson K.L., Yan L.L., Rathouz P.J., Hulley S.B., Sidney S. et al Objectively measured sleep characteristics among early-middle-aged adults the CARDIA study. American journal of epidemiology. 2006; 164(1), pp.5–16. 10.1093/aje/kwj199 16740591

[34] De Koninck J., Lorrain D., and Gagnon P. Sleep positions and position shifts in five age groups: an ontogenetic picture. 1992; 5(2):143–9. 10.1093/sleep/15.2.143 1579788

[35] Liao, W.H. and Yang, C.M. Video-based activity and movement pattern analysis in overnight sleep studies. In Pattern Recognition. 2008. ICPR 2008. 19th International Conference on (pp. 1–4). IEEE.

[36] Marshall J.D. and Nazaroff W.W. Intake fraction. Exposure Analysis. 2006 pp.237–251.

[37] Nazaroff W.W. Inhalation intake fraction of pollutants from episodic indoor emissions. Building and Environment. 2008; 43(3), pp.269–277.

